# Repetitive transcranial magnetic stimulation may be a cost-effective alternative to antidepressant therapy after two treatment failures in patients with major depressive disorder

**DOI:** 10.1186/s12888-022-04078-9

**Published:** 2022-06-28

**Authors:** Antal Zemplényi, Judit Józwiak-Hagymásy, Sándor Kovács, Dalma Erdősi, Imre Boncz, Tamás Tényi, Péter Osváth, Viktor Voros

**Affiliations:** 1 Centre for Health Technology Assessment and Pharmacoeconomic Research, Faculty of Pharmacy, University of Pécs, Rákóczi street 2, Pécs, H-7623 Hungary; 2 Institute for Health Insurance, Faculty of Health Sciences, University of Pecs, Pécs, Hungary; 3 Department of Psychiatry and Psychotherapy, Medical School, University of Pecs, Pecs, Hungary

**Keywords:** Cost-effectiveness analysis, Repetitive transcranial magnetic stimulation, Major depressive disorder, Treatment-resistant depression

## Abstract

**Background:**

The cost-effectiveness of treatment strategies for patients with Major Depressive Disorder (MDD) who have not responded to two adequate treatments with antidepressants (TRD) are still unclear. The aim of this analysis was to evaluate the cost-effectiveness of add-on repetitive Transcranial Magnetic Stimulation (rTMS) compared with standard treatment.

**Methods:**

A Markov-model simulated clinical events over one year from the perspective of healthcare payer. Third- and fourth-line treatment pathways (augmentation, antidepressant switch or combination, and Electro-Convulsive Therapy (ECT)) were defined based on medical practice guidelines. Transition probabilities were derived from a recent meta-analysis and scientific publications. Resource utilization and cost estimates were based on the patient-level database of a large university hospital.

**Results:**

Incremental Quality-Adjusted Life Years (QALYs) and costs were 0.053 and 785 €, respectively, corresponding to an Incremental Cost-Effectiveness Ratio (ICER) of 14,670 € per QALY. The difference in cost between standard treatment and rTMS is explained by the rTMS sessions used in acute (€660) and maintenance (€57/month) treatments, partly offset by lower hospital costs due to higher remission rates in the rTMS arm. Key parameters driving the ICER were incremental utility of remission, unit cost of rTMS treatment and remission rate. At a threshold of €22,243 add-on rTMS is a cost-effective alternative to pharmacotherapy. Evidence on long-term effectiveness is not yet available, so results are estimated for a one-year period.

**Conclusion:**

Not only does rTMS treatment have beneficial clinical effects compared with drug therapy in TRD, but it also appears to offer good value-for-money, especially in centres with larger numbers of patients where unit costs can be kept low.

**Supplementary Information:**

The online version contains supplementary material available at 10.1186/s12888-022-04078-9.

## Introduction

Major Depressive Disorder (MDD) is a major public health issue worldwide [1]. Depressive disorders affect nearly one-fifth of the population, the lifetime prevalence in women can be as high as 25% [2]. In 2004, depressive disorders were already the 3rd leading cause of burden in terms of Disability-Adjusted Life Years (DALYs), which are the sum of Years Lived with Disability (YLD) and Years of Life Lost (YLL) [3]. Furthermore, projections suggested that by 2020 and 2030, MDD and related suicide may be the 2nd and even the 1st leading cause of disability, respectively [1, 3, 4]. Consistent with international data, Hungarian studies found the lifetime, 1-year, and 1-month prevalence of MDD in the adult population as 15.1, 7.1, and 2.6%, respectively [5].

Early detection and effective management of depression is a priority for public health. High rates of suicide mortality among untreated depressed patients, and the chronic nature of depression can lead to absenteeism (missed days from work) or presenteeism (reduced productivity at work), work disability and significant socio-economic burden [6, 7]. Health-economics studies have shown that the direct costs of treating depression are much less than the costs and social harm caused by untreated depression [4]. Furthermore, according to a recent cost-analysis, the ratio of direct and consequent indirect costs related to the treatment of depression is 8 and 92%, respectively [5].

There are several effective psycho-pharmacological and psycho-therapeutic options to treat MDD. However, approximately 50-60% of patients with MDD do not show an adequate response to treatment or fail to achieve remission [8, 9]. Treatment-resistant depression (TRD) is most defined as an unsatisfactory response to two adequate trials of two different classes of antidepressants at the optimum dosage for a sufficient duration [10]. The proportion of TRD among MDD is between 4 and 20% (8.3% in Hungary) based on the literature and prescription data [5, 7]. Furthermore, TRD is associated with a poorer quality of life (QoL) and high economic burden [5].

In addition to pharmacotherapy, there are several new therapeutic options that may lead to remission in patients who do not respond adequately to conventional treatment. These include repetitive Transcranial Magnetic Stimulation (rTMS), a neuro-modulation technique that has now proven to be an effective and safe method in the treatment of certain mental disorders, especially in MDD and TRD [11]. rTMS may be an alternative therapy for patients who do not respond adequately to currently available psycho-pharmacotherapy or when those medications are not recommended or contraindicated. Furthermore, unlike Electro-Convulsive Therapy (ECT), rTMS does not impair cognitive functions, moreover it may even improve cognitive symptoms in MDD [12]. According to different recent guidelines (CANMAT, CTMS, NICE, WFSBP), rTMS is recommended in MDD after one or two failed antidepressant treatments as monotherapy or in combination with antidepressants in the acute phase and for maintenance treatment as well [10, 13–17].

In summary, depressive disorders, especially TRD have a significant health-economic burden [7]. Literature suggests that rTMS may be an effective and safe alternative to pharmacotherapy and ECT for those patients with MDD, who do not respond or only partially respond to conventional antidepressant treatment [14]. Furthermore, several studies have already implicated the cost-effectiveness of rTMS treatment in MDD and TRD [18, 19].

However, the availability of rTMS for patients with MDD and TRD is limited in many European countries, as it is still not reimbursed and therefore has limited access in public health systems [20]. Health technology assessment (HTA) has become a standard policy tool for informing decision makers who manage the entry and use of new technologies through reimbursement. HTA uses economic evaluations to determine the value-for-money of technologies. The aim of our analysis was to evaluate the cost-effectiveness of rTMS compared with the standard therapy (pharmaco-therapy and/or ECT) for the treatment of patients with MDD who have failed to respond at least two adequate courses of antidepressant treatment.

## Methods

### Target population

The patient population studied in the economic evaluation consists of TRD patients, who are defined as patients with MDD, who have not responded adequately to two different classes of antidepressant therapies at the appropriate dose and for the appropriate duration, and therefore require third-line treatment. The average age of the patients was considered based on the age distribution presented in a study on TRD patients [5].

### Setting and location

A cost-utility analysis was performed for evaluating interventions in the Hungarian health care context.

### Study perspective

The cost of implementing the interventions is derived from a health care sector perspective. This includes both costs of the health insurance fund and the cost of pharmaco-therapy paid by patients. Investment cost of procuring the rTMS device was not included in the base case, as it is not paid by the insurance fund. However, this was analysed in different scenarios.

### Comparators

Two treatment arms were compared in the model. The first is the standard third-line therapy of MDD, which can be an antidepressant (switch), an antidepressant adjunction or combination, an antidepressant-antipsychotic combination, and ECT treatment. The other is the technology under investigation, which is rTMS treatment in addition to antidepressant therapy. Following successful rTMS therapy, i.e., when the patient is in partial or complete remission, maintenance rTMS treatment may be used for relapse prevention.

### Time horizon

The time span of the analysis is one year, which is consistent with the typical time span for the course and treatment of acute major depression [5]. Treatment with rTMS is thought to have a beneficial effect on the relapse and also on the recurrence of depression [21, 22], however, having no long-term clinical evidence, yet we did not extrapolate the effectiveness of rTMS treatment beyond one year. Due to the one-year time horizon, it was not necessary to apply a discount rate.

### Choice of health outcomes

The health outcomes of each intervention are evaluated in Quality-Adjusted Life Years (QALYs), that was determined with a simulation model using utility values for different health conditions of patients with MDD.

### Measurement of effectiveness

Clinical effectiveness for the comparator arm, i.e., standard treatment, were taken from the STAR*D trial [23], where health conditions were defined according to Quick Inventory of Depressive Symptomatology (QIDS-SR16). Partial remission was defined as a QIDS-SR16 score reduction of at least 50% from the start of treatment, remission as a QIDS-SR16 score ≤ 5, and relapse as a QIDS-SR16 score ≥ 11, respectively. Effectiveness of ECT was derived based on Alves’ publication (2016) [24]. For the treatment arm under investigation data synthesis-based estimates were derived. A self-reported meta-analysis was conducted of randomised controlled trials (RCTs) including sham treatment control group (rTMS vs. sham control) published in the literature (S1 Supplementary material). To assess the effect of rTMS on response and remission rates, study results were synthesised using random-effects models, which accounts for possible data heterogeneity.

A targeted systematic literature review was conducted to identify utility data that matches the population and health states used in this analysis. The study [25] that was used in the model involved 307 patients with MDD receiving rTMS therapy. 92.8% of patients had recurrent depression and 43.6% had been hospitalised for depression in their history. Patients involved, in their current acute episode, had 2.5 antidepressant therapies in the past. Utilities were measured with EQ-5D at baseline and after treatment.

### Estimating resources and costs

Resource utilizations were derived from the electronic medical records of the University of Pécs and a national claims database (PULVITA), while unit costs were determined based on the reimbursement tariffs of the National Health Insurance Fund in Hungary. As rTMS treatment is not yet reimbursed in Hungary, the actual cost of treatment was estimated with microcosting method. Costs were converted to EUR based on the average exchange rate in 2020 (1 EUR = 360 HUF) [26].

### Model concept

**Fig. 1:**
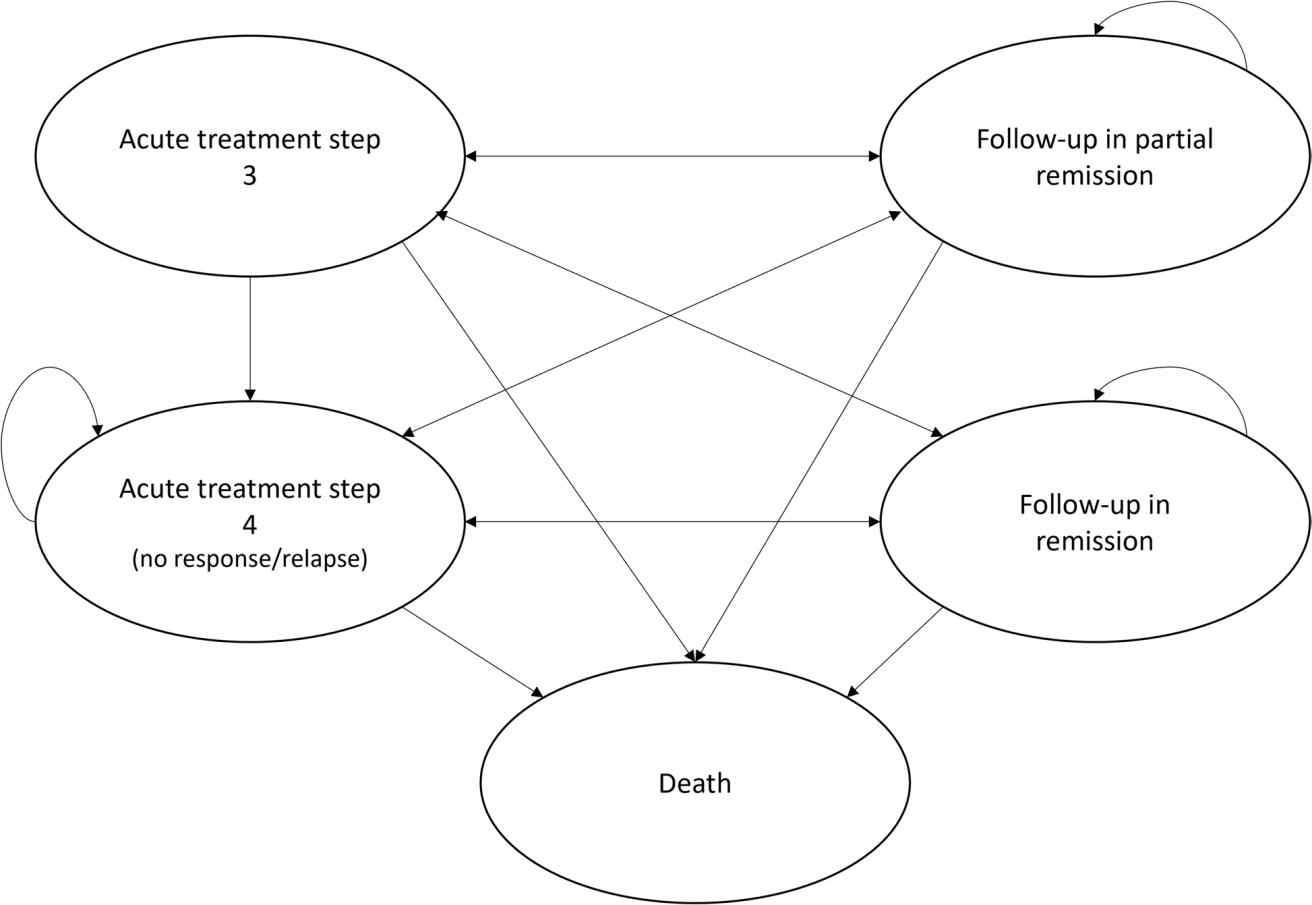
Markov-model structure for the health states and the transitions in the cost-effectiveness analysis

A Markov-simulation model was developed using TreeAge Pro 2020 software. Health states in the model were defined as: 3rd line therapy for acute depression, 4th line therapy for acute depression, partial remission, remission, and death state. The health states used in the model and the transitions are shown in Fig. 1. A more detailed representation of the model is presented in S2 Supplementary material.

In the health economic model, 2-month cycles and a half-cycle correction were applied. The results of the health economic analysis were obtained after 100,000 runs of the Markov-simulation model.

### Transition probabilities

To ensure that the cost-effectiveness model reflects the Hungarian clinical practice as closely as possible, the structure of the model, the therapeutic pathways, the patient population, and the range of therapies used were developed based on the expert opinion of medical practitioners of two leading national institutions, the Department of Psychiatry and Psychotherapy of the University of Pécs and the Department of Psychiatry and Psychotherapy of Semmelweis University.

The probabilities for the treatment decisions were derived in two steps involving expert opinions of psychiatric specialists (S3 Supplementary material): 1) a survey filled out by 14 psychiatrists was used elicit physicians’ usual treatment decisions for MDD patients who do not respond adequately to drug treatment, and 2) a consensus meeting was held to validate the model structure and patient pathways and to confirm the decision probabilities. The experts were selected based on their experience in treating MDD and TRD represented six leading psychiatric institutions in Hungary. The answers of the survey were weighted by the average number of patients in the clinics and hospitals. Probabilities defined as the weighted average of the responses to the questionnaire were included in the cost-effectiveness model. The questions and answers based on the consensus are presented in S3 Supplementary material. The probabilities of mortality are based on international [27, 28] and national literature sources [5].

### Assumptions used in the model

The key assumptions used in the model were:

- The therapeutic lines used in the STAR*D study [23] adequately reflect the practice of care in Hungary, so the effectiveness shown in this study is a good starting point for the local patient population.
- None of the treatments result in serious adverse events that would result in significant additional costs or a substantial reduction in QoL, and therefore the impact of treatment of adverse events on health gain or costs is not included in the model.
- Due to the lack of appropriate data the probability of depression-specific mortality was assumed to be the same for all conditions, regardless of whether the patient’s condition has improved or not.
- There is no learning curve or centre effect to be expected, as the professional implementation of the therapy is preceded by training of physicians and assistants, the cost of which is not borne by the hospital or health insurance budget.

### Sensitivity analysis

The effect of uncertainty in the input parameters of the health economic model on the outcome of the analysis was tested using one-way deterministic sensitivity analysis. Results are presented in the form of a tornado diagram. The effect of a +/− 10% variance for most of the parameters was tested. 

A probabilistic sensitivity analysis was performed to assess the uncertainty in the cost-effectiveness analysis by varying model assumptions simultaneously. Considering statistical distributions and standard errors, one thousand model runs were performed with the simulation of 1000 patients in each run to determine 1000 potential cost-effectiveness ratios. These results were used to evaluate the robustness of the model.

For other, general, and more detailed methodological considerations S1, S2 and S3 Supplementary materials are referred.

## Results

### Study parameters

Table 1 shows the use of health service resources and cost for rTMS, hospitalization, ECT and drug therapy.

**Table 1:** Estimated costs used in the cost-effectiveness analysis of rTMS add-on treatment versus standard therapy

Cost item	Resource use	Unit cost	Cost	SD (± 20%)	Distribution
rTMS treatment (acute treatment cost)			**660,4 EUR**	**67,4 EUR**	**gamma**
*assistant hour (25 × 1 hour)*	25	13,4 EUR	336,0 EUR		
*specialist hour (5 × 1 hour)*	5	24,7 EUR	123,3 EUR		
*office hours*	25	0,5 EUR	13,3 EUR		
*rTMS device use (sessions)*	25	6,2 EUR	154,4 EUR		
*native MR scan with (50% probability)*	50%	59,9 EUR	29,9 EUR		
*standard EEG with (50% probability)*	50%	7,0 EUR	3,5 EUR		
rTMS maintenance cost per month			**56,6 EUR**	**5,8 EUR**	**gamma**
*assistant hour (2 × 1 hour per month)*	2	13,4 EUR	26,9 EUR		
*specialist hour (2X20 min per month)*	0,66	24,7 EUR	16,3 EUR		
*office hours (per month)*	2	0,5 EUR	1,1 EUR		
*rTMS device use (sessions per month)*	2	6,2 EUR	12,4 EUR		
Hospitalization (DRG based) 40% probability	**40%**	**945,5 EUR**	**378,2 EUR**		
Cost of ECT (DRG based)	**1,00**	**945,5 EUR**	**945,5 EUR**		
Drug therapy cost per month			**13,5 EUR**	**1,4 EUR**	**gamma**
*antidepressant switch*	47%	8,0 EUR	3,8 EUR		
*antidepressant combination*	30%	17,6 EUR	5,3 EUR		
*antidepressant & antipsychotic combination use*	23%	19,5 EUR	4,5 EUR		

Table 2 shows the probability parameters and the utilities used in the simulation model.

**Table 2:** Input parameters of the cost-effectiveness model of rTMS add-on treatment versus standard therapy

Description	Base case	Min	Max	SD (± 20%)	Distribution	Source
Probabilities for treatment decisions						
getting ECT in no response condition in step 3	0,1	0,08	0,12	0,0102	beta	survey (S3)
getting ECT for the 1st time in no response condition in step 4	0,25	0,20	0,30	0,0255	beta	survey (S3)
getting ECT repeatedly in no response condition in step 4	0,05	0,04	0,06	0,0051	beta	survey (S3)
starting maintenance in step 3	0,65	0,52	0,78	0,0663	beta	survey (S3)
retreatment with rTMS after relapse if there was no maintenance rTMS therapy	0,79	0,63	0,95	0,0806	beta	survey (S3)
retreatment with rTMS after relapse when there was a maintenance rTMS therapy	0,78	0,62	0,94	0,0796	beta	survey (S3)
getting hospitalized during acute episode	0,4	0,32	0,48	0,0408	gamma	survey (S3)
Transition probabilities						
relapse after partial remission from drug therapy in step 3	0,614	0,49	0,74	0,0627	beta	[23]
relapse after partial remission from drug therapy in step 4	0,64	0,51	0,77	0,0653	beta	[23]
relapse after remission from drug therapy in step 3	0,25	0,20	0,30	0,0255	beta	[23]
relapse after remission from drug therapy in step 4	0,426	0,34	0,51	0,0435	beta	[23]
relapse after maintenance rTMS	0,173	0,14	0,21	0,0177	beta	[29]
relapse after no maintenance rTMS	0,494	0,40	0,59	0,0504	beta	[29]
remission drug therapy step 3	0,137					[23]
remission drug therapy step 4	0,13					[23]
remission when receiving ECT	0,581					[24]
remission with rTMS in step 3	0,363	0,181	0,728	0,1394	beta	meta-analysis (S1)
remission with rTMS in step 4	0,345	0,172	0,690	0,1323	beta	meta-analysis (S1)
partial response when using drug therapy step 3	0,168					[23]
partial response when using drug therapy step 4	0,163					[23]
partial response after ECT	0,262					[24]
Risk ratios						
RR of remission with rTMS	2,65	1,32	5,31			meta-analysis (S1)
RR of partial response after rTMS	1					assumption
Utilities						
incremental utility of partial remission	0,13	0,10	0,17	0,0179	gamma	[25]
incremental utility of remission	0,26	0,22	0,29	0,0179	gamma	[25]
baseline utility of no response	0,56					[25]

### Cost-effectiveness

The results of the simulation model show that the cost of rTMS treatment to the health system is higher (€2702) than conventional treatment (€1917). The additional cost of €785 is the result of multiple effects. While the implementation of rTMS treatment involves frequent face-to-face visits and requires significant human resources in health care, the higher remission rate will result in less need for hospitalisation. A higher QALY is achieved with rTMS treatment (0.658) than with standard therapy (0.605). The incremental QALY of 0.053 is mainly explained by the fact that, on average, patients spend more time in the higher utility remission health state. The incremental cost per 1 QALY of gain (incremental cost-effectiveness ratio - ICER) is €14,670 (Table 3). To determine whether this is worth public funding, it needs to be compared with the cost-effectiveness threshold used by reimbursement decision-makers.

**Table 3:** Incremental cost-effectiveness of the rTMS add-on treatment

Health technology	Annual		Incremental		ICER (€/QALY)
	Cost (€)	QALY	Cost (€)	QALY	
rTMS therapy	2702	0.658	785	0.053	14,670
Standard treatment	1917	0.605	–	–	

Hungary uses multiple threshold based on added clinical value, that is measure with Incremental Relative QALY Gain $\left(\mathrm{IRQG}=\frac{QALY_{new\ technology}-{QALY}_{comparator}}{QALY_{new\ technology}}\right)$. For technologies with IRQG less than 0.25, the applicable threshold is 1.5 times the GDP per capita, which is currently €22,243. Therefore, rTMS was shown to be cost-effective compared with the standard treatment for patients with MDD who have failed to respond at least two adequate courses of antidepressant treatment.

### Uncertainty

**Fig. 2:**
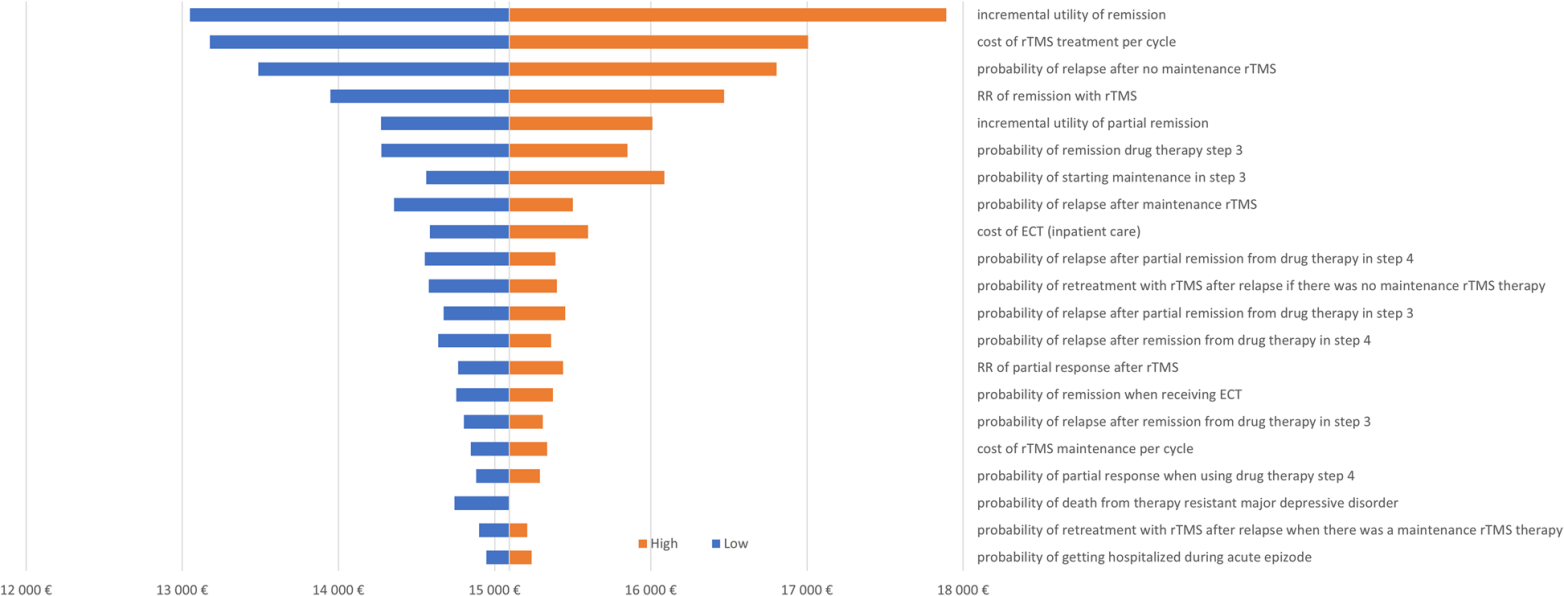
Result of the deterministic sensitivity analysis

The deterministic sensitivity analysis showed that the ICER is most sensitive to the incremental utility of remission. Other variables with substantial impact included the cost of rTMS, the probability of relapse in case no maintenance therapy was applied, and the risk ratio of remission with rTMS treatment. 10% change of any parameters would still result in a cost effectives scenario (see Fig. 2.).

**Fig. 3:**
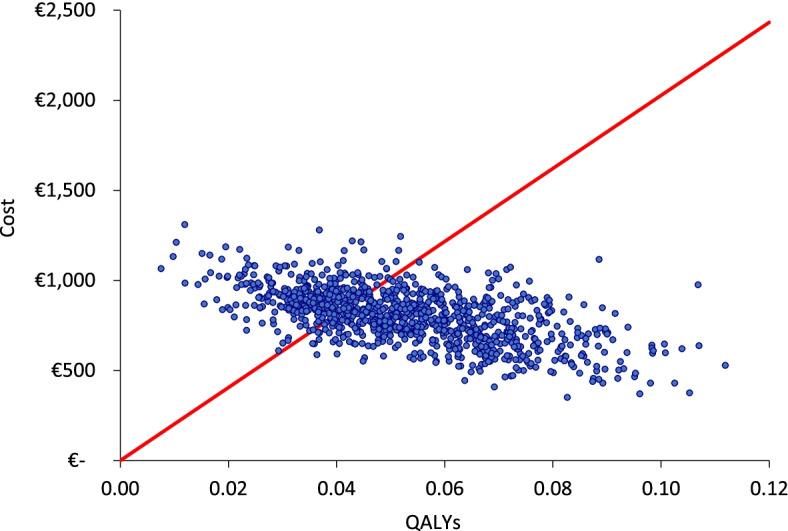
Cost-effectiveness plane (add-on rTMS vs. standard of care)

A probabilistic sensitivity analysis was conducted where the probability of rTMS being cost-effective was evaluated. Based on the meta-analysis, for the probability of remission, the limits of the 95% confidence interval of the RR of rTMS [1.32-5.31] were used. This gives a relatively broad range for the simulation, which explains the wide spread of the results. The analysis quantified that rTMS had a 70% probability of being cost-effective. The results of the probabilistic sensitivity analyses are presented in Fig. 3.

### Scenario analyses

Three alternative scenarios were examined to see how some different but realistic assumptions would affect the ICER. In the base case, it was assumed that approximately 65% of patients would receive rTMS maintenance therapy. In scenario 1, maintenance rTMS therapy was used in all patients who responded adequately to acute rTMS therapy. Since the amortisation is not financed by the health insurance fund, but the investment costs must be borne by the government or the hospital, the impact of including the costs for the amortisation of the rTMS device was investigated in scenario 2. In Hungary, there are some hospitals that do not use ECT at all for patients with MDD. Therefore, the standard treatment in scenario 3 did not include ECT, but only antidepressant pharmacotherapy. For all three scenarios, the ICERs were below the Hungarian cost-effectiveness threshold. The results of the scenario analysis are shown in Table 4.

**Table 4:** The results of the scenario analysis

Scenarios	rTMS treatment		Standard treatment		Incremental		ICER
	Cost	QALY	Cost	QALY	Cost	QALY	
Base case	2702	0,658	1917	0,605	785	0,053	14,670
Scenario 1: 100% rTMS maintenance	2672	0,668	1945	0,605	727	0,063	11,534
Scenario 2: Including amortization	2990	0,658	1950	0,605	1040	0,053	19,628
Scenario 3: without ECT	2570	0,648	1733	0,595	837	0,053	15,786

## Discussion

rTMS treatment is more expensive than drug therapy, mainly due to the human resources involved. However, with a higher number of cases in the facilities, unit costs can be kept low and thus rTMS may be a cost-effective alternative to standard therapies.

Several therapeutic alternatives may be considered in the third and fourth treatment lines during the standard care for TRD, including different pharmacotherapies and ECT, which are decided by physicians based on different guidelines and clinical practice. These were all considered as a basket of comparators in the analysis, and their utilization and unit cost were assessed accordingly.

One challenge in describing patient trajectories in the treatment of depression is that the clinician may try different new interventions depending on the response to therapy. To account for this more accurately, the simulation model considered how the likelihood of a therapy being used would change considering previous decisions. Based on the structured responses of a broad panel of experts, we could incorporate relevant decision-making practices into a patient simulation model to obtain a better estimate of the ICER.

There have been several analyses published [19, 30–39] in the literature on the cost-effectiveness of rTMS for patients with MDD who have had at least one antidepressant therapy or have TRD. In these articles, ECT was used as a comparator to rTMS treatment in nine cases and drug therapy in two publications. In most of the studies, rTMS therapy was found to be a cost-effective choice compared with either drug or ECT, but in one study in Singapore [32], one in Iran [35] and one in Spain [36], ECT was found to be more cost-effective.

In our analysis, we applied a more conservative approach compared with the two other models that used pharmacotherapy as a comparator [19, 38]. We considered it necessary to examine the cost-effectiveness of rTMS as an add-on therapy to be in line with the usual clinical practice. Nguyen et al. [19] did not delineate in their meta-analysis whether rTMS was an add-on or monotherapy, therefore their population was heterogeneous. Voigt et al. [38] defined the target population as patients with MDD who failed a pharmacotherapy trial, whereas our criterion was two failed treatments. Voigt conducted the analysis over a lifetime, while we limited the time horizon to one year, as in our judgment there were insufficient long-term clinical data on relative effectiveness.

A rapid assessment conducted by EUnetHTA in 2017 concluded that rTMS is relatively effective compared to sham treatments, with a pooled risk ratio for remission rate of 2.16 (95% CI 1.42-3.29, *p* = .0003) [20]. Nevertheless, the quality of the evidence was rated as moderate or low. Since then, other studies were published showing more robust results on the clinical effectiveness of rTMS [40]. According to our results, add-on rTMS was significantly more effective than sham rTMS, which is consistent with previous meta-analyses, but our synthesised effect size is slightly smaller than what was reported in previous studies. The meta-analysis presented here includes the results of the most recent studies and provides a more consistent and reliable conclusion on the relative effectiveness of rTMS as add-on therapy in the third line and therefore on the cost-effectiveness of this procedure.

The time horizon of our model is one year, which is consistent with the typical time span for the course and treatment of acute major depression, and which was also used in the STAR*D analysis [5, 23]. We have chosen this time period because the review of the literature also implicated, that the risk of relapse significantly drops down after a year [41, 42]. Furthermore, two years of depressive symptoms would refer rather to dysthymic disorder and not to MDD, and three years’ time period may include recurrent new depressive episodes, which are not assessed in this analysis [41]. In contrast to other previous models that have looked at three years [19] or lifetime [38], we believe that one year is an appropriate time period for analysis. If evidence of improvement in recurrence becomes available in the future, this model can be used to refine the cost-effectiveness analysis, by linking the acute episodes as a sequence.

Our analysis has some other limitations. The sample size of the studies reporting evidence on the efficacy of rTMS are low. Furthermore, due to lack of standardized protocols of rTMS interventions, substantial methodological heterogeneity exists. Thus, for the included studies there was significant variability in motor threshold, number of sessions, number of impulses and regime of maintenance. Nevertheless, the synthesized evidence in a meta-analysis showed a conclusive benefit for rTMS and the inclusion of these moderators in a mixed-effects model did not have a significant effect on the results. The other limitation is that due to the lack of evidence on long-term effectiveness, the effects of the treatment could not be extrapolated. However, rTMS may be considered cost-effective even throughout one current, acute Major Depressive Episode (MDE). Based on literature data [19, 38] and on this analysis, it can be assumed, that rTMS may have beneficial cost-effectiveness on the longer-term as well.

Furthermore, this approach may be considered as a more conservative compared with the previous analyses. In this model, the costs of rTMS may be even overestimated by counting with more specialists’ and assistants’ working hours and by applying standard rTMS sessions. With considering more recent rTMS protocols, such as theta-burst, treatment time and working hours may be significantly reduced. Furthermore, shorter treatments provide the possibility of treating more patients, and a higher number of patients in a facility may keep unit costs low. Costs can be also lower, when using rTMS as monotherapy, however remission rates may also differ in this case. Considering all these above, the real-life costs of rTMS treatment may be even lower in the future, thus the cost-effectiveness of rTMS may be more advantageous than defined by this analysis.

## Conclusion

MDD, especially TRD not only increase the suffering of patients and their relatives, but also represent a significant social and economic burden due to the rising costs of psychiatric treatment and the loss of work. Improving the efficiency of health spending and the growing burden of mental illness, especially in the current era, make the demonstration of cost-effectiveness evidence extremely important in the European countries, especially with more limited health resources. While previous health economics analyses come from US [38] and Australia [19], in the Central and Eastern European countries with lower budgets, it is even more important that funding is used for truly cost-effective interventions, such as rTMS.

## Supplementary Information

**Additional file 1: S1 Supplementary material.** Synthesizing evidence on the efficacy of rTMS by using meta-analysis.

**Additional file 2: S2 Supplementary material.** Detailed representation of the health economic model.

**Additional file 3: S3 Supplementary material.** Results of the survey and the consensus meeting.

## Data Availability

The data analysed during this study are included in this published article and its supplementary information files.

## Abbreviations

DALY: Disability-Adjusted Life Years
ECT: Electro-Convulsive Therapy
HTA: Health Technology Assessment
ICER: Incremental Cost-Effectiveness Ratio
MDD: Major Depressive Disorder
QALY: Quality-Adjusted Life Years
rTMS: repetitive Transcranial Magnetic Stimulation
TRD: Treatment-Resistant Depression
