# Supplementary material for: Repetitive transcranial magnetic stimulation may be a cost-effective alternative to antidepressant therapy after two treatment failures in patients with major depressive disorder
Source: BMC Psychiatry. 2022 Jun 28;22:437. doi: 10.1186/s12888-022-04078-9 (PMC9238085; doi:10.1186/s12888-022-04078-9)
Supplement: Supplementary file 1 — Additional file 1: S1 Supplementary material. Synthesizing evidence on the efficacy of rTMS by using meta-analysis. [file 12888_2022_4078_MOESM1_ESM.docx]

**S1 Supplementary material: Synthesizing evidence on the efficacy of rTMS by using meta-analysis**

**Methods**

The literature search began with a review of previously published meta-analyses that examined the efficacy of rTMS in Major Depressive Disorder (MDD) and Treatment-Resistant Depression (TRD). Because the most recent meta-analysis was published in 2015, a targeted literature search was conducted in the PubMed and Scopus databases covering the period 2010-2020 to include more up-to-date information in the evidence synthesis.

Evidence was synthesized from articles where the number of previous drug therapies reached or exceeded two (>=2) and an add-on study design was used, i.e. rTMS treatment was used as an adjunction to drug therapy.

The meta-analysis used a random effect model and inverse variance weighting to calculate the relative risk (RR) of remission and partial remission in the treated patient group compared with the sham-treated patient group. The main outcome of the analyses is the combined effect size and its associated confidence interval (95% CI), which can be used for pooled analyses of studies with low heterogeneity and can be interpreted as a difference using statistical methodology. Meta-analysis was conducted by using Meta-Essentials, a freely available excel-based tool (Suurmond et al., 2017; van Rhee et al., 2018).

**Results**

**Literature search**

Two reviews were found that included meta-analyses (Berlim et al., 2013; Nguyen & Gordon, 2015), which analysed 29 and 25 studies, respectively, with a total of 33 publications with significant overlap. Of these, 3 publications were excluded because the definition of health conditions did not match the conditions relevant to our analysis, and 13 articles were excluded for not meeting the criteria of augmentation or the number of previous drug therapies (>=2), resulting 17 publications to be included.

After deduplication of the results of the literature search, a total of 260 publications were found in the two databases. After screening publications by title and abstract, 54 articles were found, while at the end of the full-text review process, 10 articles were found that matched the inclusion criteria. Based on the review of previous meta-analyses (17) and database-based literature search (10), a total of 20 eligible articles were identified after filtering out overlaps.

The model used in the health economic analysis uses two health states (remission, partial remission) for patients who respond to treatment. Therefore, to follow the concept of the model, we had to subtract the number of patients in remission from the number of patients in response as reported in the articles to determine the number of patients in partial remission. Of the 20 publications selected, only 10 contained sufficient data to perform this transformation. For this reason, the data from these 10 articles were finally considered for meta-analysis and their result was used as an input parameter in the model. Their data are presented in Table 1.

1. Table: Summary table of data from the 10 articles included in the meta-analysis

| **First author, year** | **Hz** | **MT** | **N of sessions** | **N of pulses** | **Applied depression**  **scale** | **Randomized double blinded sham controlled** |
| --- | --- | --- | --- | --- | --- | --- |
| **Filipčić, 2019** | 10 Hz | 120% | 20 | 60000 | HAMD-17, MADRS | Y |
| **Taylor, 2018** | 10 Hz | 120% | 20 | 60000 | MADRS, HRSD, QIDS-SR, GAD-7 | Y |
| **Blumberger, 2016** | 10 Hz | 120% | 15 | 31500 | HAMD | Y |
| **Bakim, 2012** | 20Hz | 80 % and 110% | 30 | 24000 | HAMD-17, MADRS | Y |
| **Blumberger, 2012** | 10 Hz | >100% | 15 | 21750 | HDRS | Y |
| **Rossini, 2005a** | 15 Hz | 80% and 100% | 10 | 6000 | HAMD, CGI-S, CGI-I | Y |
| **Su, 2005** | 5 Hz, 20 Hz | 100% | 10 | 16000 | HAMD, HAM-A, CGI-S, BDI | Y |
| **Koerselman, 2004** | 20Hz | 80% | 10 | 8000 | HAMD | Y |
| **Boutros, 2002** | 20Hz | 80% | 10 | 8000 | HAMD | Y |
| **Padberg, 2002** | 10 Hz | 90% and 100% | 10 | 15000 | MADRS, HRSD | Y |

**Results of the meta-analysis**

**Remission**

The heterogeneity of the data from the included studies is low (pQ=0.228, I2=23.36), so the combined outcome measure and its statistical test and the resulting confidence interval are meaningful. The RR of entering remission status in the group of patients receiving rTMS treatment was 2.65 (95% CI: 1.37-5.31, p=0.002), as shown in Table 2 and Figure 1.

2. Table: Summary table of results of meta-analyses

|  | **Response** | **Remission** | **Partial Response** |
| --- | --- | --- | --- |
| **Combined Effect Size** | | | |
| **Risk Ratio** | 2.32 | 2.65 | 1.13 |
| **CI Lower limit** | 1.61 | 1.32 | 0.51 |
| **CI Upper limit** | 3.32 | 5.31 | 2.52 |
|  |  |  |  |
| **Z-value** | 5.26 | 3.16 | 0.34 |
| **One-tailed p-value** | 0 | 0.001 | 0.367 |
| **Two-tailed p-value** | 0 | 0.002 | 0.734 |
|  |  |  |  |
| **Number of incl. subjects** | 603 | 603 | 603 |
| **Number of incl. studies** | 10 | 10 | 10 |
| **Heterogeneity** | | | |
| **Q** | 8.21 | 11.74 | 11.11 |
| **p_Q_** | 0.514 | 0.228 | 0.268 |
| **I^2^** | 0.00% | 23.36% | 19.01% |
| **T² (Risk Ratio)** | 0 | 0.24 | 0.28 |
| **T (Risk Ratio)** | 0 | 0.49 | 0.53 |

1. Figure: Forest plot on the effectiveness of rTMS treatment (remission)


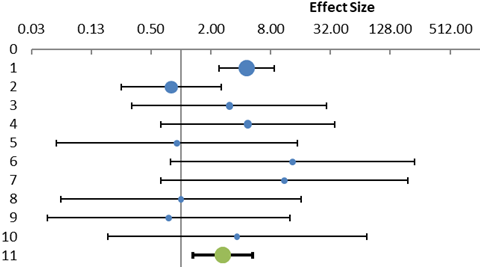


**Partial remission**

The heterogeneity of the data from the included studies among these articles is also low (pQ=0.268, I2=19.01%), so the combined outcome measure and its statistical test and the resulting confidence interval are meaningful. The RR of being in partial remission, according to the requirements of the health economic model used, in the group of patients receiving rTMS treatment was 1.13 (95% CI: 0.51-2.52, p=0.734), as shown in Table 2 and Figure 2.

2. Figure: Forest plot on the effectiveness of rTMS treatment (partial remission)


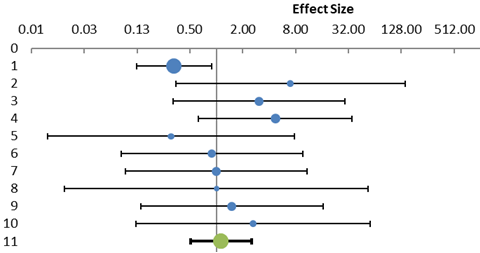


In the health economic model, the partial and full remission probabilities from the STAR*D trial were multiplied by the relative improvement (RR) derived from the meta-analysis. As the difference in RR of rTMS treatment for partial remission is not statistically significant, but rTMS was found to be more favourable in terms of response (see Table 2), a conservative approach was taken and the RR of partial remission in the two study arms was considered equal in the cost-effectiveness model.
