# Supplementary figures and images for: Repetitive transcranial magnetic stimulation may be a cost-effective alternative to antidepressant therapy after two treatment failures in patients with major depressive disorder
Source: BMC Psychiatry. 2022 Jun 28;22:437. doi: 10.1186/s12888-022-04078-9 (PMC9238085; doi:10.1186/s12888-022-04078-9)

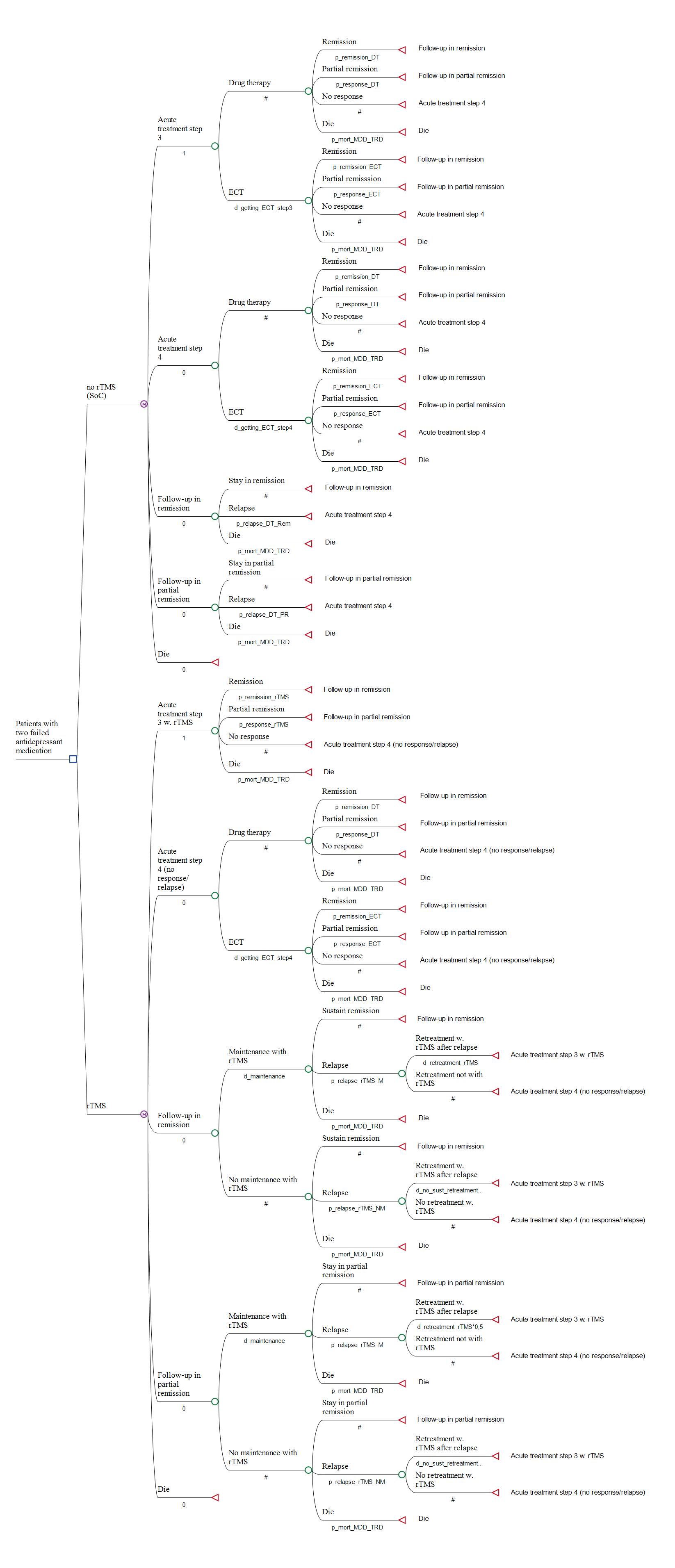

Supplement: Supplementary file 2 — Additional file 2: S2 Supplementary material. Detailed representation of the health economic model. [file 12888_2022_4078_MOESM2_ESM.png]
