# Supplementary material for: Repetitive transcranial magnetic stimulation may be a cost-effective alternative to antidepressant therapy after two treatment failures in patients with major depressive disorder
Source: BMC Psychiatry. 2022 Jun 28;22:437. doi: 10.1186/s12888-022-04078-9 (PMC9238085; doi:10.1186/s12888-022-04078-9)
Supplement: Supplementary file 3 — Additional file 3: S3 Supplementary material. Results of the survey and the consensus meeting. [file 12888_2022_4078_MOESM3_ESM.docx]

**S3 Supplementary material: Results of the survey and the consensus meeting**

Three authors of the manuscript are national opinion-leader psychiatrists (TT, PO, VV), who actively participated in the development of the psychiatry-specific parts of the cost-effectiveness model.

However, in order to ensure that the cost-effectiveness model reflects global national clinical practice as closely as possible, further consultations were held with representatives of the domestic psychiatric profession:

1. Data collection was accomplished through a questionnaire survey on the clinical practices of treating patients with major depressive disorder (MDD) and treatment-resistant depression (TRD), who have not responded adequately to the treatment as usual (TAU).
2. A consensus meeting was held to validate the model structure, patient pathways, treated patient population and treatment alternatives.

Hungarian psychiatric experts from the 5 leading institutions in the country were asked to participate in the survey to validate the structure and results of the model from the following institutions:

- Semmelweis University, Department of Psychiatry and Psychotherapy (SU)
- National Institute of Mental Health, Neurology and Neurosurgery, Nyírő Gyula Hospital (NIMH)
- University of Pécs, Department of Psychiatry and Psychotherapy (UP)
- University of Szeged, Szent-Györgyi Albert Clinical Center, Department of Psychiatry and Psychotherapy (USz)
- University of Debrecen, Kenézy Gyula University Hospital, Department of Adult Psychiatry (UD)

**I. RESULTS OF THE SURVEY**

The following questions were asked about the treatment of patients with MDD and TRD:

- In case of MDD patients who have not responded adequately or have been resistant to treatment, how often do you currently use the following drug therapy options:
  - Switch of medication?
  - Augmentation?
  - Combination?
- In case of MDD patients who have not responded adequately or have been resistant to treatment, what is the likelihood of applying Electro-Convulsive Therapy (ECT):
  - After two unsuccessful treatment attempts?
  - After three unsuccessful treatment attempts?
  - After more unsuccessful treatment attempts ?
- In case of MDD patients who have not responded adequately or have been resistant to treatment, what is the likelihood of applying repetitive Transcranial Magnetic Stimulation (rTMS):
  - As maintenance treatment if the patient has previously been in remission with TMS treatment?
  - Again, as an acute treatment, if
    - Relapse develops after a successful acute TMS treatment followed by maintenance TMS treatment?
    - No maintenance TMS treatment is applied following a successful acute TMS treatment and a relapse occurs?

The answers were weighted by the average number of patients in the clinics and hospitals. Weights were based on the number of patients discharged from each psychiatric ward in 2019. Probabilities defined as the weighted average of the responses to the questionnaire were included in the cost-effectiveness model. Questionnaire responses are summarised in Table 1.

1. Table: Results of the survey

| **INSTITUTE** | **Total number of patients discharged from psychiatric wards in 2019** | **WEIGHT*** | **In case of MDD patients who have not responded adequately or have been resistant to treatment, how often do you currently use the following drug therapy options:** | | | **In case of MDD patients who have not responded adequately or have been resistant to treatment, what is the likelihood of applying electroconvulsive treatment:** | | | | | | **In case of MDD patients who have not responded adequately or have been resistant to treatment, what is the likelihood of applying transcranial magnetic stimulation:** | | |
| --- | --- | --- | --- | --- | --- | --- | --- | --- | --- | --- | --- | --- | --- | --- |
|  |  |  |  |  |  | **after two unsuccessful** treatment attempts (one antidepressant and one rTMS) | | **after three unsuccessful treatment** attempts (two antidepressants and one rTMS) | | **after more unsuccessful** treatment attempts (e.g. antidepressant, rTMS, ECT), if | | **as maintenance treatment if the patient has previously been in remission beside TMS treatment?** | **again as an acute treatment, if** | |
|  |  |  | Change of medication (AD to new AD) | Augmentation  (beside AD the use of another drug (lithium or antipsychotics) | Combination  (beside AD a new AD) | if the patient has had a partial response (partial remission) | no response (relapse) | if the patient has had a partial response (partial remission) | no response (relapse) | the patient has had a partial response (partial remission) | no response (relapse) |  | relapse develops after a successful acute TMS treatment followed by maintenance TMS treatment? | no maintenance TMS treatment is applied following a successful acute TMS treatment and a relapse occurs? |
| **UP** | 1.339 | 11% | 32.5% | 32.5% | 36.3% | 0.625% | 0.125% | 1.875% | 3.125% | 1.25% | 2% | 51.25% | 32.5% | 59.125% |
| **NIMH** | 4.205 | 36% | 57.5% | 20% | 22.5% | 15% | 15% | 25% | 27.5% | 37.5% | 40% | 50% | 75% | 75% |
| **USz** | 1.325 | 11% | 50.0% | 20% | 20% | 0% | 0% | 50% | 50% | 50% | 50% | 100% | 100% | 50% |
| **UD** | 2.135 | 18% | 30.0% | 20% | 50% | 10% | 10% | 20% | 20% | 5% | 5% | 90% | 70% | 90% |
| **SU** | 2.683 | 23% | 50.0% | 25% | 25% | 30% | 50% | 40% | 60% | 50% | 80% | 60% | 100% | 100% |
| **NUMBER OF PATIENTS-WEIGHTED PROBABILITY** | **11.687** | **100%** | **47%** | **23%** | **29%** | **14%** | **19%** | **28%** | **33%** | **32%** | **40%** | **65%** | **78%** | **79%** |

*Weights were based on the number of patients discharged from each psychiatric ward in 2019.

Source: National Health Insurance Fund of Hungary: Hospital bed and patient flow report 2019.

**II. RESULTS OF THE CONSENSUS MEETING**

Experts of University of Pécs, Department of Psychiatry and Psychotherapy and Semmelweis University, Department of Psychiatry and Psychotherapy validated the model structure, patient pathways, treated patient population, treatment alternatives and the results of the survey in a consensus meeting. The questions and answers based on the consensus are presented in Table 2.

2. Table: Results of the consensus meeting

| **QUESTIONS** | **CONSENSUS ANSWERS** |
| --- | --- |
| What is a health technology-enabled procedure? How many times will the treatment be given? | rTMS treatment 5 times a week for 4-6 weeks in combination with medical treatment |
| What are the indications? | Treatment of major depressive disorder (MDD) |
| What patient population is it applied to? | Patients with MDD, who have not responded adequately to two different antidepressant therapies |
| What is considered standard therapy for the given indication and patient population? | Medication (psycho-pharmacological treatment), ECT |
| Which therapies would the new health technology replace or complement? | Medication (psycho-pharmacological treatment), ECT |
| Health conditions relevant for MDD treatment? | Remission, partial remission, no response, death |
| Do the patient pathways in the model represent clinical practice? | Based on the model structure, yes |
| After 2 unsuccessful AD treatments, what percentage of patients receive medication and ECT therapy? | ECT: 10%  Medication: 90% |
| After 3 unsuccessful AD, what percentage of patients receive medication and ECT therapy? | ECT:25%  Medication: 75% |
| After 2 unsuccessful AD and 1 unsuccessful ECT, what percentage of patients receive medication and ECT therapy? | ECT: 5%  Medication: 95% |
| After 2 unsuccessful AD treatments and 1 unsuccessful rTMS treatment, what percentage of patients receive medication and ECT therapy? | ECT: 25%  Medication: 75% |
| What percentage of patients receiving 3rd line treatment in the acute phase are hospitalised? | 40% of patients receiving 3rd line medical treatment are hospitalised |
| Are 100% of patients receiving ECT treatment reported and accounted for a DRG code - diagnosis-related group, in Hungarian HBCs - (Depressive syndromes: 822A,B)? | 100% of patients receiving ECT are treated as in-patients |
